# Impact of the delay to start treatment in patients with lung cancer treated in a densely populated area of Brazil

**DOI:** 10.6061/clinics/2017(11)05

**Published:** 2017-11

**Authors:** Fernando Conrado Abrao, Igor Renato Louro Bruno de Abreu, Roberto Odebrecht Rocha, Felipe Dourado Munhoz, João Henrique Godoy Rodrigues, Riad Naim Younes

**Affiliations:** I Departamento de Cirurgia Toracica, Faculdade de Medicina Santa Marcelina, Sao Paulo, SP, BR; II Departamento de Cirurgia Toracica, Centro de Oncologia do Hospital Alemao Oswaldo Cruz, Sao Paulo, SP, BR

**Keywords:** Lung Neoplasms, Public Health, Mortality

## Abstract

**OBJECTIVES:**

The aim of this study is to evaluate the access of patients with lung cancer in a densely populated area of São Paulo to the Brazilian Public Health System, focusing on the time spent from symptom onset or initial diagnosis until the beginning of treatment.

**METHODS:**

We retrospectively reviewed 509 patients with malignant lung neoplasms who were admitted to a single reference oncology center of the public health system between July 2008 and December 2014. Patients were considered eligible for this study if they were older than 18 years and had not undergone any previous oncology treatment when they were admitted to the institution. The following data were collected from all patients: age, gender, smoking status, tumor staging, time from the when the first symptoms were experienced by the patient to when the patient was diagnosed with cancer, time from the first appointment to cancer diagnosis, and time from when the patient was diagnosed with cancer to the initiation of treatment.

**RESULTS:**

The median time from symptom onset to diagnosis was three months. From the first appointment to diagnosis, the median time interval was one month; however, 79% of patients were diagnosed in up to two months. The median time from diagnosis to the start of treatment was one month, but most patients (82.5%) started treatment in up to two months.

**CONCLUSION:**

In our highly populated region with preferential access to the public health system, patients are required to wait a relatively long time to effectively begin treatment for lung cancer. This type of study is important to alert medical societies and government health agencies.

## INTRODUCTION

Lung cancer is the malignant neoplasm with the highest incidence worldwide 1,2. In developed countries, the incidence has decreased in men and remained stable among women, reflecting a reduction in the rate of smoking in males 3. Lung cancer is also the leading cause of cancer-related deaths worldwide, with an estimated 1.1 million deaths in men and 427,400 in women in 2012 4. These numbers are greater than the sum of deaths due to breast, prostate and colon cancers and account for approximately 18% of deaths from all cancers 1.

In 2016 in Brazil, 17,490 new cases of lung cancer were estimated in men and 10,540 in women. These values correspond to an estimated risk of 16.79 new cases per 100,000 men and 10.75 per 100,000 women. Excluding non-melanoma skin tumors, lung cancer in men is the second most common malignancy in the southern (35.17 / 100,000) and midwestern (14.53 / 100,000) regions of Brazil. In the southeastern (19.02 / 100,000), northeastern (9.75 / 100,000) and northern (8.07 / 100,000) regions of Brazil, lung cancer is the third most common cancer among men. For women, lung cancer is the third most common malignancy in the southern (20.61 / 100,000) and southeastern (10.56 / 100,000) regions. In the midwestern (9.37 / 100,000) and northeastern (7.24 / 100,000) regions of Brazil, lung cancer is the fourth most common malignancy in women. In the northern region (5.07 / 100,000), lung cancer is the fifth most common among women 5. Unlike the data from developed countries, the lung cancer incidence and mortality in Brazil follow an upward curve, with progressively higher diagnostic and mortality rates. 6. In 1992, Pereira et al. described the profile and access of lung cancer patients in the Brazilian Public Health System (SUS). The author showed data related to the initial staging and warned about the delay in the diagnostic process 7. However, this study did not assess the necessary time to start treatment. After this study, the literature over the past few decades did not include another study that assessed the evolution of health care access of lung cancer patients in the SUS.

The aim of this study is to evaluate the recent access (in the last six years) of lung cancer patients to the SUS, including the time required to start treatment.

## METHODS

This study is part of a research project approved by the Ethics Review Committee at our institution, registered under protocol 49258615.4. We retrospectively reviewed 509 patients with malignant lung neoplasms who were admitted to a single reference oncology center of the public health system between July 2008 and December 2014, which were our inclusion criteria. The median follow-up time was seven months (range 1 – 77 months). Our institution is one of the reference oncology centers in the state of São Paulo, which is the most densely populated area of Brazil. Our center is recognized by the executive board of the public health system as an institution able to treat any type of cancer, including pediatric cancer.

Patients diagnosed with lung cancer during the study period were identified by the Cancer Registry database of our institution. Additional clinical information on individual patients was collected retrospectively from medical records kept at our institution. All patients admitted were originally from public health care units (general practitioners or pulmonologists) and required a recommendation to the oncology center from the executive board of the public health system. The board of the public health system refers these patients to one of the reference oncology centers. Patients were considered eligible for this study if they were older than 18 years and had not undergone any previous oncology treatment when they were admitted. All 509 patients were eligible for this study. We did not find any patients who had undergone previous oncology treatment or patients younger than 18 years old. Histopathological exams were registered to confirm lung cancer diagnoses. All patients admitted to our institution without histopathological exams were submitted to tumor biopsy guided by computed tomography (CT), ultrasound or bronchoscopy. Patients with suspected stage I lung cancer were subjected to a CT scan that included the thorax, abdomen and pelvis. Patients with suspected stage II or more advanced disease were subjected to positron emission tomography (PET) and brain nuclear magnetic resonance imaging. Patients who were candidates for surgical treatment were subjected to a frozen biopsy of the lung tumor and the mediastinal lymph nodes in the operating room. The following data were collected from all patients: age range, gender, smoking status and tumor staging according the American Joint Committee on Cancer and the International Union for Cancer Control update of the tumor-node-metastasis (TNM) cancer staging system 8,9. In this study, we also evaluated the influence of time until diagnosis and time to initiate the treatment on the survival of patients with lung cancer. The data were collected and arranged in the following time intervals:

- Time (months) from the date when the first symptoms were experienced by the patient (patient history, PH) to the date when the patient was diagnosed with cancer (DX); we excluded 37 patients who were asymptomatic when we conducted this specific analysis.
- Time (months) from the date of the first appointment (first app) with a specialist (oncologist or thoracic surgeon) to the date when the patient was diagnosed with cancer (DX); we excluded seven patients from whom we did not recover this information when we conducted this specific analysis.
- Time (months) from the DX date to the starting date of treatment (TTO).

### Statistical Analyses

Descriptive analysis of the data (i.e., distribution and the presence of outliers) was carried out using measures of central tendency (median and interquartile range), measures of dependence (correlation and covariance) and measures of dispersion (amplitude and/or standard deviation) for continuous variables and frequency for categorical variables. Categorical and continuous variables were compared by Fisher’s exact test or by chi-squared and Mann-Whitney U tests, respectively. In addition, measures of skewness and a coefficient of kurtosis were evaluated.

Death related to lung cancer was the principal outcome evaluated in this study. Kaplan-Meier survival estimates were used to determine the five-year lung cancer-specific survival for all patients, and the log-rank (Mantel-Cox method) and Breslow (generalized Wilcoxon) analyses were used to compare the differences between factors. The cutoff values were chosen based on medical literature regarding delayed treatment in lung cancer 10-12. Survival was calculated from the date of patient admission to our institution to the date of the last follow-up or until death from any cause. The data are presented as the medians and 95% confidence intervals (CIs; lower – upper bounds).

Univariate and multivariate Cox proportional hazard analyses were performed to investigate the prognostic significance of the demographic and predictive risk factors on the survival outcome of patients with lung cancer. Statistical analyses were performed using SPSS version 17.0 for Windows. A two-tailed value of p < 0.05 indicated statistical significance.

## RESULTS

The demographic characteristics of the 509 lung cancer patients are summarized in Table 1. The mean age of these patients was 62.5 years (standard deviation: 11.2 years), and there was a slight predominance of male (57.8%) over female (42.2%) patients. More than 75% of these patients were smokers. Lung adenocarcinomas (37.3%) and squamous cell carcinoma (28.7%) were the most common types of tumors observed in the patients, and carcinoid tumors (2.1%) were the least frequent type. Regarding clinical stages, most patients (68.3%) came to our hospital when the disease was already at an advanced stage (stages III and IV). Most of these patients began receiving treatment at up to 1.5 months after DX (Table 2). Approximately one-fifth of the patients were eligible for surgery (20.2%), and lobectomy (67.0%) was the most frequently performed surgical procedure. For all patients, including those who received no cancer-specific treatment, the median overall survival was seven months (95% CI: 5.7 to 8.2 months), with 34.5% of these patients surviving for one year and 8.1% surviving for five years.

Patients spent a relevant amount of time waiting during each interval period. For instance, the median time from PH to DX was 3 months. From the first app to DX, the median time interval was 1 month; however, 79% of patients were diagnosed in up to 2 months. Finally, the median time from DX to TTO was 1 month (range: 0 – 17 months), but most patients (82.5%) started treatment in up to 2 months.

**Figure 1:**
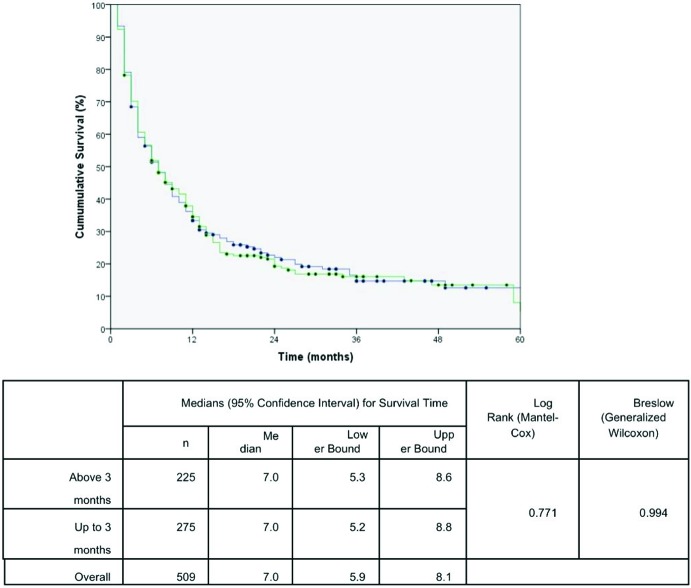
Kaplan Meier curve for mortality due to lung cancer in patients according to time from the patient history (PH) to diagnosis (DX). The blue line represents the patients diagnosed up to 3 months, and the green line represents patients diagnosed above 3 months.

**Figure 2:**
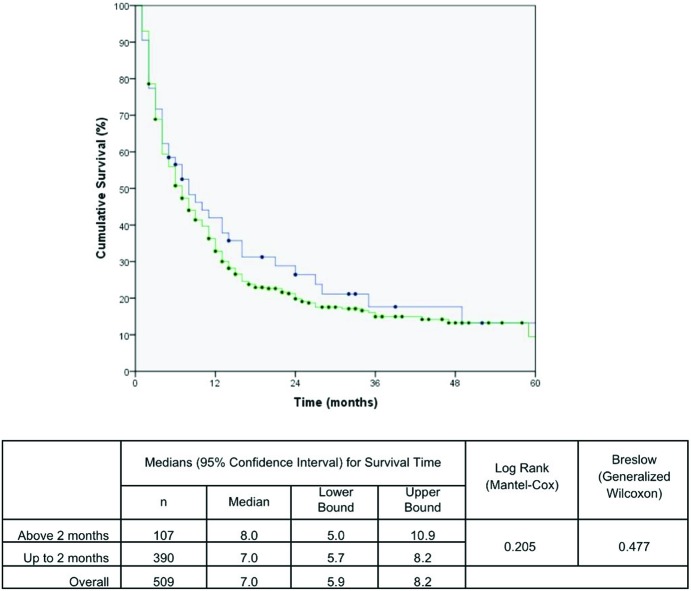
Kaplan Meier curve for mortality due to lung cancer in patients according to time from the appointment (first app) to diagnosis (DX). The blue line represents the patients diagnosed up to 2 months and green line above 2 months.

**Figure 3:**
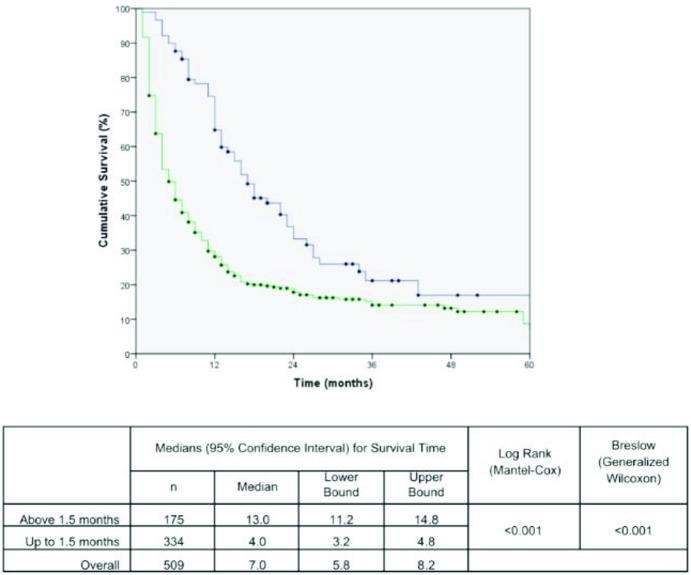
Kaplan Meier curve for mortality due to lung cancer in patients according to time from diagnosis (DX) to the starting date of treatment (TTO). The blue line represents the patients that started treatment above 1.5 months and green line up to 1.5 months.

The effects of the time intervals from the development of the first symptoms to TTO on lung cancer-associated mortality are shown in the set of Kaplan-Meier survival curves using the log-rank and/or Breslow statistical tests. A Kaplan-Meier curve demonstrated a significant negative impact on the cumulative five-year survival curve for patients who started treatment up to 1.5 months after DX. The time from PH to DX and the time from the first app with the general practitioner to DX were not statistically significant (Figures 1, 2 and 3). These results were confirmed in the multivariate analysis (Table 3).

## DISCUSSION

In Brazil, to the best of our knowledge, one study has assessed the time from diagnosis to treatment of lung cancer 7. In 1992, Pereira et al. studied patients at all stages and not only patients who were candidates for surgical resection 7. Our study data, obtained 23 years later, suggest that the time required for lung cancer diagnosis has decreased. The data also suggest that diagnosis is faster when the patient can make an appointment with a specialist (i.e., a pulmonologist, thoracic surgeon or oncologist). Additionally, as far as we know, this is the first study that evaluated the time to begin cancer treatment or palliative care across all cancer stages in the public health system. Other positive results on the survival from this study are the cancer stage, histological type of lung cancer and surgical treatment. However, these results are well stablished in the medical literature.

Pereira et al. reported that only 44.3% of patients were diagnosed with lung cancer within 90 days. On the other hand, 45.5% of patients were diagnosed after 120 days. However, the same study showed that access to the public health system was fast, with 88% of patients arriving at the first consultation within 30 days after symptom onset, but only 10% were initially seen by a specialist in the onco-pulmonology area.

Our study showed that the median time from PH to DX was 90 days, and 79% of cases had a lung cancer diagnosis confirmed within 60 days. Another important finding is that after the first consultation with a specialist, the median time to diagnosis was 30 days. These data suggest that in the last 23 years, there was a decrease in the time to diagnosis of lung cancer and that medical specialists, not generalists, played an important role in accelerating the time to diagnosis. In the United States, Nadpara et al. reported a median time between first symptomatic presentation and diagnosis of approximately 180 days 13. A median diagnostic interval of 113 days (with an upper quartile value of 249 days) was reported in a recent English primary care records study of lung cancer patients diagnosed between 2007 and 2010 14.

However, regarding the time required to start treatment after diagnosis, 50% of patients required up to 30 days, 32.5% required between 30 and 60 days, and 17.5% required more than 60 days. On the other hand, in South Korea, Shin et al. described only the surgical delay for patients with lung cancer, and the median time from cancer diagnosis to surgery was 20 days 15. In Finland, for all patients with lung cancer, the median time from cancer diagnosis to treatment was 15 days 10. Our study did not assess the possible causes that could explain our results. However, it is noteworthy that the number of patients who waited more than 30 days to start treatment is substantial.

Regarding the decrease in survival observed in patients who started treatment up to 1.5 months after diagnosis, Myrdal et al. found the same result 11. In both studies, the number of patients in clinical stages III and IV was higher in the group treated early (Table 2). Therefore, in agreement with Myrdal et al., we believe that more severe patients, i.e., those at more advanced stages, end up being prioritized to start treatment sooner than those at lower stages. The authors of both studies believe that this phenomenon reflects the health system’s incapacity to absorb all patients. Perhaps this deficiency of the public health system could be minimized with the implementation of early detection programs for lung cancer.

Our study has several limitations. This is a retrospective study of data from a single reference oncology center. Mortality data should be interpreted with caution, as the study included patients who received exclusively palliative treatment as well as patients with different types of biological tumors (e.g., small cell carcinoma).

We conclude that there was a reduction in the time to lung cancer diagnosis over the past two decades. Access to a specialist plays a crucial role in diagnosis in the public health system, and in our densely populated area, patients have to wait a relatively long time to begin treatment. This type of study is important to alert medical societies and government health agencies.

## AUTHOR CONTRIBUTIONS

Abrao FC designed the study, analyzed the data, wrote the manuscript, reviewed the analysis of the data and approved the final version of the manuscript. Abreu IR reviewed the analysis of the data and approved the final version of the manuscript. Rocha RO analyzed the data and reviewed the statistical analysis. Munhoz FD and Rodrigues JG reviewed the analysis of the data and helped to conduct the study. Younes RN helped to design the study, analyzed the data and helped to write the manuscript. All the authors read and approved the final version of the manuscript.

## Figures and Tables

**Table 1:** Demographic characteristics of the 509 lung cancer patients.

Parameters	Total Number	%
**Patient age** *(mean±SD), years*	(62.5±11.2)	
		
**Number of patients**	509	100%
Gender		
*Male*	294	57.8%
*Female*	215	42.2%
		
**Histology type of primary tumor**		
*Adenocarcinoma*	187	37.3%
	11	2.1%
*Large cell carcinoma*	24	4.7%
*Small cell lung cancer*	74	14.5%
*Squamous cell carcinoma*	146	28.7%
*Undefined*	67	13.2%
		
**Stages of cancer**		
*Ia/Ib*	37	7.3%
*IIa/IIb*	28	5.5%
*IIIa/IIIb*	74	14.5%
*IV*	274	53.8%
*Extensive ^a^*	58	11.4%
*Limited ^b^*	15	2.9%
*Undefined*	23	4.5%
		
**Surgical procedure**		
*Yes*	103	20.2%
*No*	406	79.8%
		
**Surgical procedure type**		
*Bi-lobectomy*	1	1.0%
*Lobectomy*	69	67.0%
*Pneumectomy*	15	14.5%
*Segmentectomy*	9	8.7%
*Thoracotomy*	9	8.7%
		
**Disease recurrence**		
*Yes*	127	24.9%
*No*	382	75.1%
		
**Smoker**		
*Yes*	400	78.6%
*No*	109	21.4%
*Cigarette packs per year*		
*(mean ± SD)*	(50.5±32.9)	

a Extensive disease: Beyond the ipsilateral hemithorax, which may include malignant pleural or pericardial effusion or hematogenous metastases (TNM: T any, N any, M Ia/b; T3-T4 due to multiple lung nodules that do not fit in a tolerable radiation field; 9).

b Limited disease: Confined to the ipsilateral hemithorax, which can be safely encompassed within a tolerable radiation field (TNM: T any, N any, M 0; except T3-T4 due to multiple lung nodules that do not fit in a tolerable radiation field; 9).

**Table 2:** Stage of lung cancer with respect to time (months) from the date when the patient was diagnosed with cancer (DX) to the starting date of treatment (TTO), showing the number of patients at each stage according to the different delays*.

Stage of lung cancer	Number of patients (%)	Delay between DX and TTO ≤1.5 months	Delay between DX and TTO >1.5 months
	Total	Total	Total
	509 (100)	334 (65)	175 (35)
Ia/Ib	37	26 (70)	11 (30)
IIa/IIb	28	14 (50)	14 (50)
IIIa/IIIb	74	46 (62)	28 (38)
IV	274	177 (65)	94 (35)
Extensive	58	40 (69)	18 (31)
Limited	15	08 (53)	07 (47)
Undefined	23	22 (96)	01 (04)

* *p* value = 0.039 (Fisher’s exact test, ≤1.5 *vs*. >1.5 months).

**Table 3:** Risk factors (prevalence odds ratio, 95% CI) associated with patient death by univariate and multivariate analyses (Cox regression).

Risk factors, N (%)	Univariate analysis	Multivariate analysis	Multivariate analysis *p* value
**Age** > *62 years*	1.1 (0.9 – 1.3)	not included	
**Gender** *Male*	1.1 (0.9 – 1.4)	not included	
			
**Histology type of primary tumor**			
*Adenocarcinoma*	Comparator		
*Large cell carcinoma*	1.3 (0.8 – 2.2)		
*Squamous cell carcinoma*	1.3 (1.0 – 1.6)		
*Small cell lung cancer*	2.3 (1.7 – 3.2)*	2.0 (1.5 – 2.8)	<0.001
			
**Stages of cancer**			
*Ia/Ib*	comparator		
*IIa/IIb*	1.6 (0.8 – 3.2)	2.5 (1.4 – 4.6)	0.002
*IIIa/IIIb*	2.7 (1.6 – 4.7)*	3.0 (1.7 – 5.4)	<0.001
*IV*	3.7 (2.2 – 6.2)*	-	ns
*Extensive*	3.9 (2.2 – 6.9)*	-	ns
*Limited*	2.7 (1.3 – 5.6)*	-	ns
			
**No surgical treatment for lung cancer**	comparator		
**Surgical treatment for lung cancer**	0.4 (0.3 – 0.6)*	0.6 (0.4 – 0.9)	0.005
**Time from PH to DX** > *3 months*	1.0 (0.8 – 1.2)	not included	
**Time from first app to DX** *> 2 months*	1.2 (1.0 – 1.5)	not included	
**Time from DX to TTO** *< 1.5 months*	1.8 (1.4 – 2.2)*	2.1 (1.6 – 2.6)	<0.001

* *p* value <0.05 (univariate analysis by Cox regression).
